# Gender Difference of Alanine Aminotransferase Elevation May Be Associated with Higher Hemoglobin Levels among Male Adolescents

**DOI:** 10.1371/journal.pone.0013269

**Published:** 2010-10-12

**Authors:** Solomon Chih-Cheng Chen, Jun-Jun Yeh, Mei-Hwei Chang, Yu-Kuei Liao, Li-Chen Hsiao, Choo-Aun Neoh, Teck-Siang Tok, Jung-Der Wang

**Affiliations:** 1 Department of Pediatrics, Pingtung Christian Hospital, Pingtung, Taiwan; 2 Department of Internal Medicine, Pingtung Christian Hospital, Pingtung, Taiwan; 3 Department of Pediatrics, National Taiwan University Hospital, Taipei, Taiwan; 4 Department of Community Health, Pingtung Christian Hospital, Pingtung, Taiwan; 5 Department of Public Health, National Cheng Kung University College of Medicine, Tainan, Taiwan; 6 Departments of Internal Medicine, Environmental and Occupational Medicine, National Cheng Kung University Hospital, Tainan, Taiwan; 7 Institute of Occupational Medicine and Industrial Hygiene, National Taiwan University, Taipei, Taiwan; Aga Khan University, Pakistan

## Abstract

**Background:**

To explore the gender difference of ALT elevation and its association with high hemoglobin levels.

**Methods:**

A cross-sectional study of 3547 adolescents (2005 females, mean age of 16.5?.3 years) who were negative for hepatitis B surface antigen received health checkups in 2006. Body mass index (BMI), levels of hemoglobin, ALT and cholesterol were measured. ALT >42 U/L was defined as elevated ALT. Elevated ALT levels were detected in 112 of the 3547 participants (3.3%), more prevalent in males than in females (5.4% vs. 1.4%, p<0.001). Hemoglobin levels had a significant linear correlation with ALT levels in both genders. Abnormal ALT started to occur if hemoglobin >11 g/dl in females or >13.5 g/dl in males, but the cumulative cases of elevated ALT increased more quickly in males. Proportion of elevated ALT increased as either the BMI or hemoglobin level rise, more apparent in male adolescents. Logistic regression modeling showed odds ratio (95% confidence interval) were 24.7 (15.0–40.6) for BMI ≥27 kg/m^2^; 5.5 (2.9–10.4) for BMI 24–27 kg/m^2^; 2.7 (1.3–5.5) for Q5 (top 20th percentile) hemoglobin level; and 2.6 (1.6–4.1) for male gender. Further separately fitting the logistic models for two genders, the significance of Q5 hemoglobin level only appeared in the males.

**Conclusions:**

High hemoglobin level is a significant risk factor of ALT elevation after control hepatitis B, obesity and gender. Males have greater risk of abnormal liver function which may be associated with higher hemoglobin levels.

## Introduction

Male gender has been noted to have a higher risk of elevated alanine aminotransferase (ALT) in previous epidemiological studies [1], [2], [3], [4], [5]. However, no clear mechanism exists to explain how sex affects the development of abnormal liver function among adolescents. In general, male adolescents have higher hemoglobin levels than those of females, because the latter regularly lose blood in the menstrual cycle. As males have both higher levels of hemoglobin and ALT than females, we are interested in the possible association between hemoglobin and ALT levels among adolescents and the potential hypothesis of the gender difference.

The aim of this study is to explore the gender difference of ALT elevation and its association with hemoglobin levels among adolescents.

## Methods

### Study Population

To facilitate early detection of morbidity, the Bureau of Health of the local government in Taiwan has an annual program that provides a health checkup for all students in the tenth grade in senior high schools. The Department of Community Health of Pingtung Christian Hospital took up the responsibility for performing these checkups in Taitung and Pingtung counties in southern Taiwan in 2006. A total of 3610 adolescents from 11 senior high schools received physical checkups between August and October of 2006. The participation rate was nearly 100%, as the health checkup was mandatory for all students. To avoid any potential confounding effect from hepatitis B infection, we have excluded 63 students with positive hepatitis B surface antigen (HBsAg), 30 males and 33 females, from the analysis. The remaining 3547 adolescents, 1542 males and 2005 females, had a mean age and standard deviation of 16.5±1.3 years.

### Laboratory Data and Definitions

Anthropometric measures and blood sampling were done after an overnight fast. Body height (BH), body weight (BW) and serum biochemistry including alanine aminotransferase (ALT), aspartate aminotransferase (AST) and cholesterol levels, were measured and recorded. All of the analyses were performed using a Beckman Coulter LX-20 autoanalyzer. In our hospital, an ALT level above 42 IU/L was defined as elevated, indicating abnormal biochemical function of the liver. A cholesterol level over 200 mg/dl was defined as abnormal. The normal ranges of hemoglobin are 12–16 g/dl for females and 14–18 g/dl for males. The hemoglobin level of each gender was classified into 5 quintiles: Q1 (0^th^ – 20^th^ percentiles), Q2 (21^st^ – 40^th^ percentiles), Q3 (41^st^ – 60^th^ percentiles), Q4 (61^st^ – 80^th^ percentiles), and Q5 (81^st^ – 100^th^ percentiles). Hepatitis B markers including hepatitis B surface antigen (HBsAg) and hepatitis B surface antibody (anti-HBs) were measured using a radioimmunoassay (Abbott Laboratory, U.S.A.) and marked as positive or negative. BMI (body mass index) was calculated in kg/m^2^ and classified into the following three categories as suggested by the Department of Health in Taiwan: normal (BMI <24 kg/m^2^), overweight (BMI ≥24 but <27 kg/m^2^) and obese (BMI ≥27 kg/m^2^) categories.

### Ethics Statement

The Ethics Review Board of our institute approved the protocol before the commencement of this study. All participants or their parents provided us with an informed consent. All data were collected for statistical analysis only, without revealing any personal identification.

### Statistical Methods

Statistical analysis was performed using SAS version 9.1 (SAS Institute, Inc., Cary, NC). A *p* value <0.05 was considered to represent a statistically significant difference. Risk factors including BMI, gender and hemoglobin levels were taken into consideration as determinants or risk factors in the construction of multivariate logistic regression models with the level of ALT as the dependent variable. Adjusted odds ratios (ORs) were estimated and 95% confidence intervals (95% CI) were also calculated.

## Results

### Basic Factors Categorized by Gender

The distributions of age, anthropometric measurements (BH, BW and BMI), hemoglobin levels, ALT, AST, cholesterol and states of hepatitis B infection of all 3547 adolescents are summarized and stratified by gender in Table 1. Compared with female adolescents, male adolescents have significantly higher values of BH, BW, and BMI, as well as higher levels of ALT, AST and hemoglobin, but lower cholesterol levels (Table 1). Elevated ALT was detected in 112 (3.2%) participants, and was more prevalent in males than in females (5.4% vs. 1.4%, *p*<0.001).

**Table 1:** Basic information including age, anthropometric measurements, biochemical data and hepatitis B antibody status of 3547 adolescents stratified by gender.

	Male	Female	*p* value
Number (%)	1542 (43.5)	2005 (56.5)	
Age (mean ± S.D.), years	16.6±1.6	16.4±1.0	0.675
Body height (mean ± S.D.), cm	170.4±6.3	159.4±5.5	<0.001
Body weight (mean ± S.D.), kg	63.8±14.4	52.7±9.8	<0.001
BMI (mean ± S.D.)	21.9±4.5	20.7±3.6	<0.001
<24, n (%)	1161 (75.3)	1724 (86.0)	<0.001
24 – 26.9, n (%)	176 (11.4)	157 (7.8)	
≥27, n (%)	205 (13.3)	124 (6.2)	
ALT (mean ± S.D.), U/L	19.2±15.3	14.4±8.7	<0.001
>42 U/L, n (%)	83 (5.4)	29 (1.4)	<0.001
≤42 U/L, n (%)	1459 (94.6)	1976 (98.6)	
Hemoglobin (mean ± S.D.), g/dl	14.8±1.1	13.1±1.3	<0.001
Cholesterol (mean ± S.D.), mg/dl	157.6±29.3	174.6±30.2	<0.001
>200 mg/dl, n (%)	116 (7.5)	354 (17.7)	<0.001
≤200 mg/dl, n (%)	1426 (92.5)	1651 (82.3)	
Anti-HBs antibody positive, n (%)	521 (33.8)	765 (38.2)	0.007
Antibody negative, n (%)	1021 (66.2)	1240 (61.8)	

Student t-test or Chi square test, with p value <0.05 as significant.

BMI  =  body mass index.

ALT  =  alanine aminotransferase.

AST  =  aspartate aminotransferase.

S.D.  =  standard deviation.

**Figure 1:**
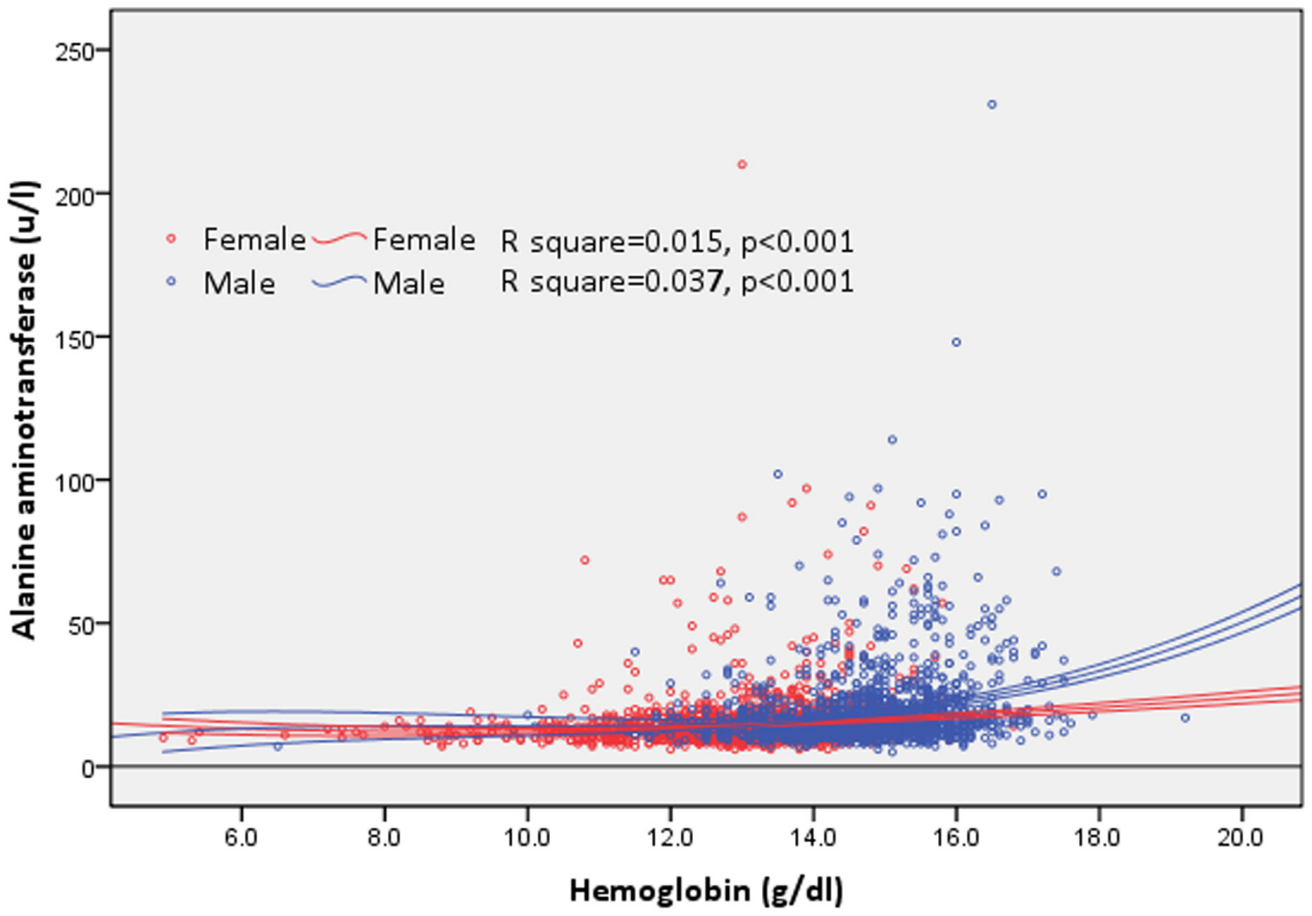
The distribution of alanine aminotransferase (ALT) levels by hemoglobin levels among 3547 adolescents. Two fitting regression lines of two genders are drawn by cubic equation with statistical significance, p<0.001.

**Figure 2:**
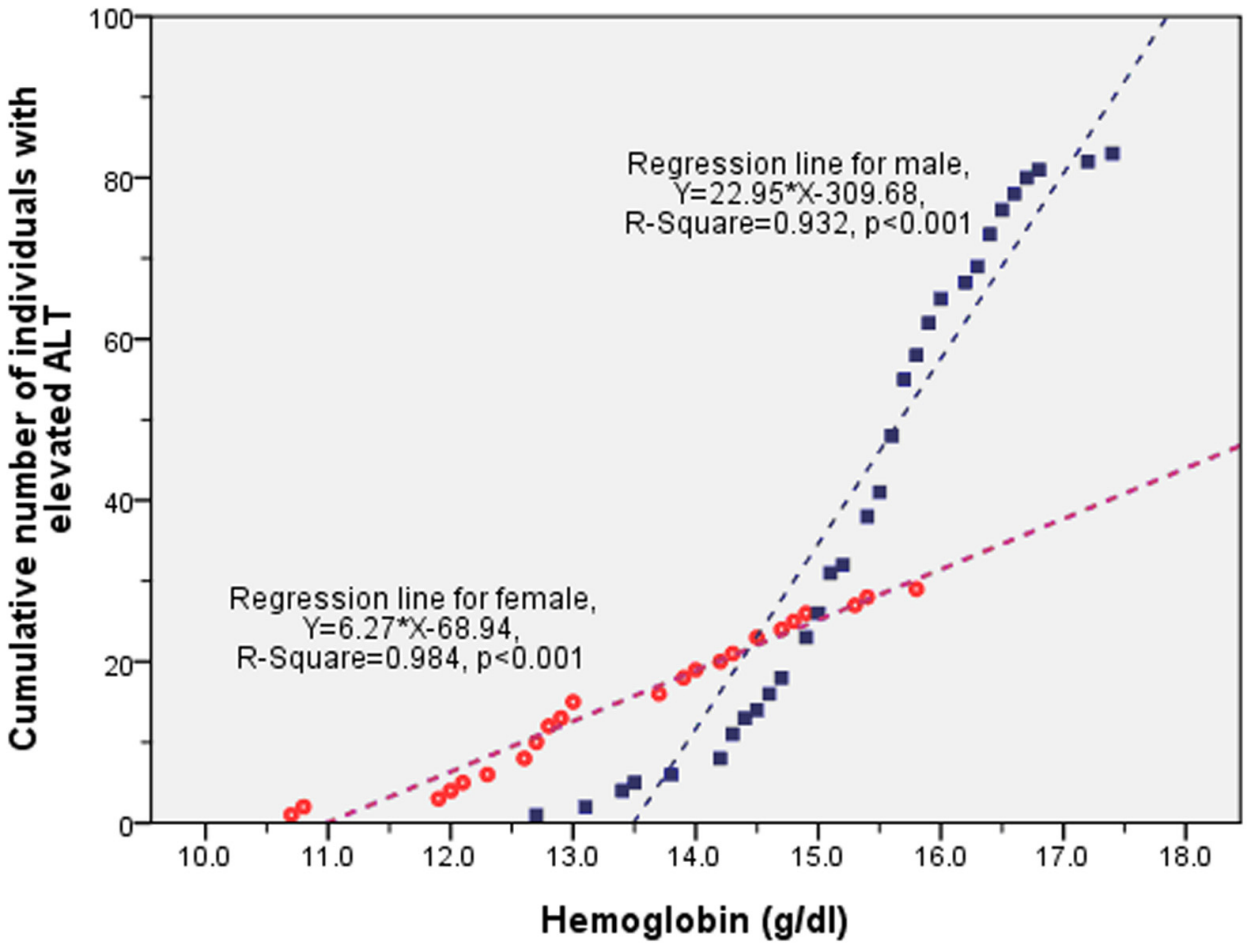
The cumulative number of individuals with abnormal ALT has a linear correlation with the hemoglobin levels for both genders, with both regression lines being statistically significant. The slope of the regression line is higher for males than for females: 22.95 vs. 6.27. According to the regression lines, the hemoglobin levels at which the cumulative number of abnormal ALT would be zero, are about 11.0 g/dl for females and 13.5 g/dl for males.

The linear correlations between the hemoglobin and ALT levels are significant for two genders (Figure 1). Furthermore, the cumulative numbers of individuals with abnormal ALT and hemoglobin levels also yield statistically significant linear correlations in both genders, with a much steeper slope for males than that of females (Figure 2). According to the regression analyses, there seems to be a threshold level for hemoglobin of which the number of subjects with abnormal ALT begins to exceed zero, and that level is about 11.0 g/dl for females and 13.5 g/dl for males.

**Figure 3:**
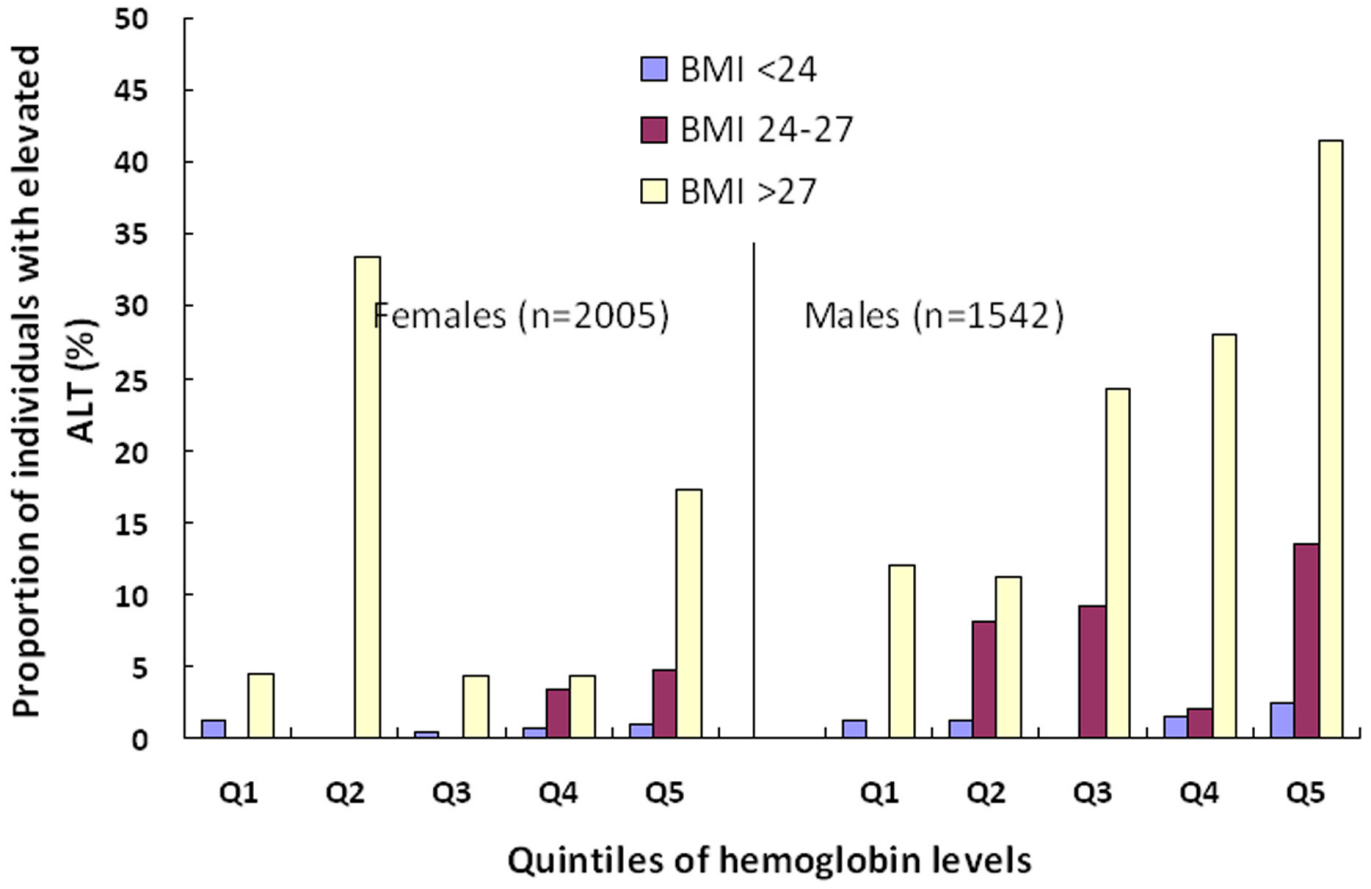
The proportion of individuals with abnormal alanine aminotransferase (ALT), categorized by body mass index (BMI) and quintiles of hemoglobin levels by gender. The total numbers of individuals with abnormal ALT are 29 and 83 cases among 2005 females and 1542 males, respectively.

When stratified by gender and BMI classification, we found that elevated ALT occurs more frequently at higher hemoglobin levels (Figure 3). There seemed to have a dose-response relationship between increased proportions of individuals with elevated ALT and increased hemoglobin levels among males, but not apparent among female adolescents.

### Risk Factors Associated with ALT Elevation

Table 2 compared the odds ratio (OR) and 95% confidence interval (95% CI) for risk factors associated with elevated ALT among three different multivariate logistic regression models. Through such comparisons, we found an effect modification by gender on the association between increased hemoglobin and elevated ALT. In other words, the OR is significant for Q5 hemoglobin level in males but not in females. In particular, the statistically significant risk factors for elevated ALT were overweight, obesity, Q5 hemoglobin level, and male gender after adjustment for all other risk factors.

**Table 2:** Comparisons of odds ratio (OR) and 95% confidence interval (95%CI) for risk factors associated with elevated alanine aminotransferase (ALT) among different logistic regression models.

	ALT, U/L>42/≤42	Model 1 = All subjectsOR (95%CI)	Model 2A = Male adolescentsOR (95%CI)	Model 2B = Female adolescentsOR (95%CI)
Gender				
Female	29/1976	1		
Male	83/1459	**2.6 (1.6–4.1)**		
Body mass index, kg/m^2^				
<24	24/2885	1	1	1
24–26.9	15/333	**5.5 (2.9–10.4)**	**5.9 (2.7–12.8)**	**4.3 (1.3–14.0)**
≥27	73/329	**24.7 (15.0–40.6)**	**25.1 (13.4–47.0)**	**24.5 (10.6–56.4)**
Cholesterol, mg/dl				
≤200	83/2994	1	1	1
>200	29/441	**1.8 (1.1–2.9)**	1.5 (0.8–2.8)	**2.4 (1.1–5.6)**
Hemoglobin, g/dl				
Q1	11/699	1	1	1
Q2	16/691	1.4 (0.6–3.1)	1.3 (0.4–3.8)	1.6 (0.5–5.0)
Q3	14/696	1.0 (0.4–2.3)	1.6 (0.6–4.5)	0.4 (0.1–1.9)
Q4	22/685	1.3 (0.6–2.8)	1.8 (0.7–4.9)	0.7 (0.2–2.6)
Q5	49/664	**2.7 (1.3–5.5)**	**3.8 (1.5–9.7)**	1.4 (0.5–4.6)

## Discussion

For adolescents, the known risk factors of elevated alanine aminotransferase (ALT) include viral hepatitis infections [6], [7], [8], obesity [8], [9], [10], [11], metabolic syndrome [12] and drugs [13]. After excluding all hepatitis B carriers, this study identifies following risk factors significantly associated with ALT elevation among adolescents, including obesity, high hemoglobin level and male gender. Since the hemoglobin level is generally higher in male adolescents than that in female adolescents (14.8±1.1 vs. 13.1±1.3, *p*<0.001, Table 1), we suspect the effect of gender difference on ALT elevation may be related to the difference of hemoglobin levels between two genders.

Obesity has been reported as a significant risk factor for liver function impairment and BMI is a good predictor of elevated ALT [9], [10], [11], [14]. The present study also corroborates the above claim among adolescents, as summarized in the logistic regression models in Table 2.

Second to obesity, high hemoglobin levels were found to be significantly associated with ALT elevation in this study. Although we found a linear correlation between ALT levels and hemoglobin levels in Figure 1, such an association becomes more apparent when the hemoglobin level is above 11.0 g/dl for females and 13.5 g/dl for males as shown in Figure 2. Further analysis by multivariate logistic regression models in Table 2 found that only Q5 (in the top 20^th^ percentile) hemoglobin levels are significantly associated with a higher risk compared to Q1 (in the bottom 20^th^ percentile), with an OR of 2.7 (95% CI, 1.3–5.5). Furthermore, there appears a dose-response relationship between increased proportion of individuals with elevated ALT and increased hemoglobin levels in Figure 3, and such a relationship is the most apparent in the category of BMI >27 kg/m^2^, followed by BMI 24–27 kg/m^2^ and the least in BMI <24 kg/m^2^. Therefore, we think both BMI and hemoglobin levels synergistically contribute to the elevation of ALT.

For gender effect, this study found a higher percentage of males with elevated ALT than that of females (5.4% vs. 1.4%, *p*<0.001, Table 1). Moreover, the slope of the correlation lines between accumulated number of individuals with elevated ALT and hemoglobin level for two genders is steeper for male adolescents than that for females (22.95 vs. 6.27 in Figure 2), indicating an effect modification by gender. Different logistic regression models in Table 2 showed the attempts to clarify the interaction between gender and hemoglobin level on ALT elevation. In the model 1 with all subjects included, both gender and Q5 hemoglobin level were significant risk factors for ALT elevation. Further analysis by separately fitting the logistic models for different genders showed that the significance of Q5 hemoglobin level only appeared in the males (Table 2), corroborating the hypothesis of a gender effect. Furthermore, the figure 3 also supports that increased BMI is a major risk factor of elevated ALT among males, but not females. In contrast, cholesterol was found to be associated with elevated ALT only for females but not males in Table 2.

At the same time, there were 63.7% (61.8% in females and 66.2% in males, *p* = 0.007) adolescents without detectable hepatitis B antibody (anti-HBs), which is consistent with previous studies in Taiwan showing approximately a 63–64% undetectable rate of anti-HBs among adolescents who were born after the implementation for the universal HBV vaccination program [15], [16], [17]. All the above facts corroborate the validity of this study.

Because hepatitis C, another important risk factor for abnormal liver function, is not endemic in Pingtung and Taitung counties of Taiwan, and none of these adolescents have ever received blood transfusion before, it did not appear to be a confounder in this study. Some infectious agents like hepatitis A, malaria, enteric fever that may also induce ALT elevation are very rare in Taiwan which is already a well-developed country with good sanitary environment. Moreover, these adolescents were apparently well before the health checkup. So, we did not think these infections would be confounders. Obstructive sleep apnea as a possible co-morbidity of bariatric patients with morbid obesity was also associated with an elevated hemoglobin [18]. Although in our daily clinical practice, sleep apnea was very rare in Taiwanese adolescents, we still carefully evaluated each subject. In total, there were only 8 participating adolescents with BMI over 40 and none of them suffered from sleep apnea. Moreover, the association between hemoglobin and elevated ALT persists after stratification by BMI categories. So, the sleep apnea cannot be a confounder for the relationship between a higher hemoglobin and elevated ALT. In addition, histories of medication, alcohol drinking, drugs or chemical exposures were evaluated in this study, and none of the participants had such a problem. Finally, as the prevalence of HFE gene mutation among Taiwanese healthy people was only 0.33% of carrier rate [19], and its association with iron overload among CHC or NAFLD patients was not significant[20], the likelihood of this alternative explanation is also minimal. Thus, we tentatively concluded that the major risk factors were captured in this study.

However, the exact mechanism by which high hemoglobin is associated with elevated ALT remains unclear. To the best of our knowledge, only one study in Japan found that hemoglobin over 14.0 g/dl was a strong predictor of ALT elevation in severely obese women [21]. Since the existence of a threshold level for increased hemoglobin seems to be associated with elevated ALT in Figure 2 as well as their dose-response relationship in Figure 3, we suspect that the higher hemoglobin level might serve as a surrogate marker for a possible overload of iron in the human body, which has been found to be associated with elevated ALT [22], [23], [24], [25], [26]. It has been approved that regular phlebotomy to remove excess body iron can improve ALT level among chronic hepatitis C cases [27], [28], [29], [30], [31], [32], aid histological recovery in patients with non-alcoholic fatty liver diseases (NAFLD) [33], [34]. Since the menstrual cycle of female adolescents is akin to a monthly phlebotomy, it may explain why female adolescents in this study have a much lower prevalence of elevated ALT (Table 1) and the effect modification by sex in the construction of multivariate logistic regression models (Table 2).

Some limitations of the present study should be mentioned. First, the number of subjects with abnormal ALT was relatively small in both males and females, since these adolescents were apparently healthy students regularly attending senior high schools and just received a routine health checkup. As the proportion was not high, it was not appropriate to draw any strong inference. Second, since smoking might be related to an increased hemoglobin and a national survey in 2007 found the smoking rate among the senior high school students were 14.0% [35], it raises the concern that smoking might be a potential confounder in this study. However, as smoking was not associated with ALT elevation in our previous study [25], and people aged less than 18 years old are not allowed to purchase cigarettes and students are forbidden to smoke in the high school in Taiwan, the likelihood of ALT elevation caused by heavy smoking among these adolescent is probably minimal. Third, this was a cross-sectional study, which precludes the determination of a causal relationship between elevated ALT level and the associated factors. Fourth, due to budget constraints, we did not perform ultrasonography to help diagnose fatty liver or NAFLD. Instead, we used elevated ALT level as a proxy indicator in our daily practice for screening.

In conclusion, the current study indicates that obesity, Q5 hemoglobin level (top 20^th^ percentile) and male gender might be associated with elevated ALT among Taiwanese adolescents. Furthermore, the significance of Q5 hemoglobin level only appeared in male adolescents, indicating the high hemoglobin level may be a potential risk factor for elevated ALT, especially for male adolescents. However, further study is needed to test the association between high hemoglobin and possible iron overload and the increased risk of elevated ALT in adolescents.
